# Optimal Self-Tuning PID Controller Based on Low Power Consumption for a Server Fan Cooling System

**DOI:** 10.3390/s150511685

**Published:** 2015-05-20

**Authors:** Chengming Lee, Rongshun Chen

**Affiliations:** Department of Power Mechanical Engineering, National Tsing Hua University, 101, Section 2, Kuang-Fu Road, Hsinchu 30013, Taiwan; E-Mail: d9533820@oz.nthu.edu.tw

**Keywords:** server fan cooling system, PID neural network, optimal self-tuning, fan power model

## Abstract

Recently, saving the cooling power in servers by controlling the fan speed has attracted considerable attention because of the increasing demand for high-density servers. This paper presents an optimal self-tuning proportional-integral-derivative (PID) controller, combining a PID neural network (PIDNN) with fan-power-based optimization in the transient-state temperature response in the time domain, for a server fan cooling system. Because the thermal model of the cooling system is nonlinear and complex, a server mockup system simulating a 1U rack server was constructed and a fan power model was created using a third-order nonlinear curve fit to determine the cooling power consumption by the fan speed control. PIDNN with a time domain criterion is used to tune all online and optimized PID gains. The proposed controller was validated through experiments of step response when the server operated from the low to high power state. The results show that up to 14% of a server’s fan cooling power can be saved if the fan control permits a slight temperature response overshoot in the electronic components, which may provide a time-saving strategy for tuning the PID controller to control the server fan speed during low fan power consumption.

## 1. Introduction

The rapid growth in information technology has contributed to the high demand for commercial servers, making power consumption by the cooling fans a major concern in server design. Currently, commercial servers are commonly equipped with multiple CPU sockets and densely housed to execute network applications. For example, high-quality Internet services (e.g., web hosting and e-commerce services) require thousands of servers to attain high-performance computing capabilities. High-density servers have a high likelihood of thermal failure, and therefore require additional power consumption for cooling. Generally, numerous fans used for cooling off the electronic components are mounted within the server in a serial or parallel manner. Lefurgy *et al*. [1] indicated that fan power can reach up to 51% of the overall server power budget. In addition, some studies [2,3] have indicated that an additional 0.5–1 W of power is required for the cooling equipment for every 1 W of power used for operating a single server. Therefore, fan power efficiency improvement attracts increasing attention as the demand for high-density servers increases.

Fan power efficiency relies on the design of the fan speed control. Recently, researchers have considered closed loop fan control schemes with numerous temperature sensors for optimizing the fan controller to save as much cooling power as possible [4,5,6,7]. The goal of a fan control is to maintain a component’s temperature at a set point. To address high fan power dissipation caused by the over-provisioning of airflow, a model-based fan controller has been proposed to achieve convex optimization [6]. However, thermal model construction requires a series of experiments for identifying parameters such as thermal resistance and related fluid properties. Furthermore, until now, studies of fan-cooling power saving have focused on determining power consumption when the server is stable at its peak power. However, power consumption in the transient response time, when the server operates from low to high power state, has not been discussed. In reality, it may take a considerable amount of time, possibly up to 10 min or more, to reach a stable temperature when the server load is changed. If the transient response performance can be optimized according to fan power consumption, considerable cooling power can be saved.

In the industry, the proportional-integral-derivative (PID) algorithm is by far the most popular feedback control method. It has a simple control structure and a clear physical meaning of three control gains, and is easy to implement [8,9,10]. The control performance of a PID controller depends on the combination of gains. To achieve more desirable control performance, the three gains must be adjusted to meet the system requirements. To date, various methods have been proposed for implementing self-tuning PID controllers [11,12]. This study focused on PID neural network (PIDNN) self-tuning because of its simple structure and nonlinear learning ability [13]. The PIDNN has been successfully employed in various applications, such as in pH neutralization and a batch reactor [14], motion control of a two-wheeled vehicle [15], a single and double inverted pendulum system [16], and a five-degrees-of-freedom active magnetic bearing system [17]. Moreover, Liu *et al*. [18] detailed the design schemes of the optimal-tuning PID controller in different industry applications, such as time domain optimal-tuning PID control, frequency-domain optimal-tuning PID control, and multiobjective optimal-tuning PID control. The schemes are chosen carefully as suitable for the system characteristics, and the optimization is based on the desired system specification.

This study aimed to reduce as much fan power consumption as possible because of the increasing demand of cooling power in servers. In addition to being easy to implement, a PID controller systematically analyzes transient performance through optimal-tuning strategies. Furthermore, the self-tuning PID controller is a convenient substitute for labor- and time-intensive manual tuning. Therefore, we propose a PIDNN self-tuning PID controller that is optimized using time domain specifications. The requirement is defined on the basis of low fan power. A server mockup system was constructed for validating the proposed method. We compared the control results in various time domain specifications of the self-tuning PID controller. The results suggested that slight overshoot of the temperature response of electronic components should be permitted when designing the fan speed control for lower fan power consumption.

## 2. Description and Problem Formulation

### 2.1. Server Mockup System

**Figure 1:**
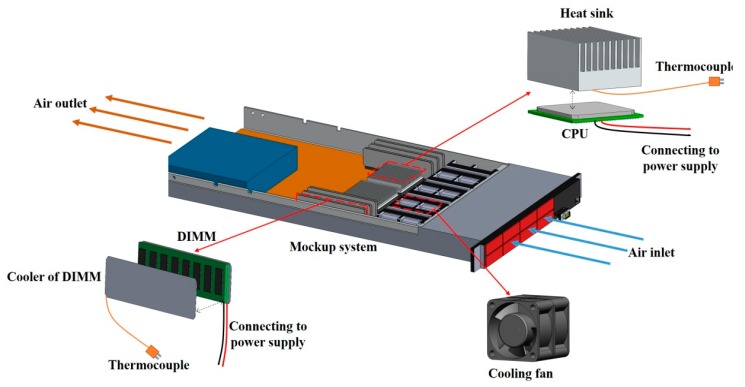
Server mockup system.

To evaluate the PID tuning technique by using neural network with time domain optimization for the server fan cooling system, a mockup system exhibiting typical characteristics of a real server was constructed. The mockup server was a 1U rack server which is often used as the basic server type for building a datacenter U, representing a rack unit, is a standardized unit describing the height of commercial servers, and one rack unit is 44.45 mm high. For example, 1U means that the height of a server is 44.45 mm, and 2U server is 88.9 mm high. As shown in Figure 1, the overall outer dimensions of the server are 670 mm (length) × 330 mm (width) × 44.5 mm (height), and the system layout and components placement play a major role in the thermal characteristics and cooling requirements. Eight axial cooling fans, which provide cooling sources, are mounted in parallel. When the pulse width modulation (PWM) signals are provided to the fans, the air travels from the inlet to the outlet of the server. The heat sources originate from the operation of the electronic components within the server including dual chip processor units (CPU) and two groups of dual in-line memory modules (DIMM). In this study, CPUs and DIMMs were heated by the power supply to represent their thermal loading in operation. To obtain the temperatures of the components, we used thermocouples, affixed on the cooler of the components, as temperature sensors for measuring various temperatures. Therefore, because the temperatures are sensed, all of the components can maintain their temperature set points through adjustment of server fans.

### 2.2. Problem Formulation

The modeling of the server fan cooling system is complex. A resistor–capacitor (RC) network [19,20] has been proposed to derive thermal and cooling models. It can be used to study the fan-based cooling power efficiency of the server. However, complex model construction hinders time-to-market server sale as well as model variations because of various server configurations. Thus, the fan-based model of the server is created to investigate the power efficiency by using fan control. Modern servers have taken advantage of the variable speeds of fans driven according to thermal limits for high fan power efficiency. Moreover, PWM is the most widely used for variable fan speed driven. In theory, fan law [21] explains that fan power consumption is proportional to a cubic function of the rotational speed of the fan rotor, expressed as follows.

(1) $$P\propto F^{3}$$

where

*P* = power, ft-lb/min
*F* = rotational speed, rpm

Thus, a third-order polynomial is adopted to represent the relationship of fan power and the rotational speed [5].

(2) $$P(F)=aF^{3}+bF^{2}+cF+d$$

where *a*, *b*, *c*, and *d* are the constant coefficients.

**Figure 2:**
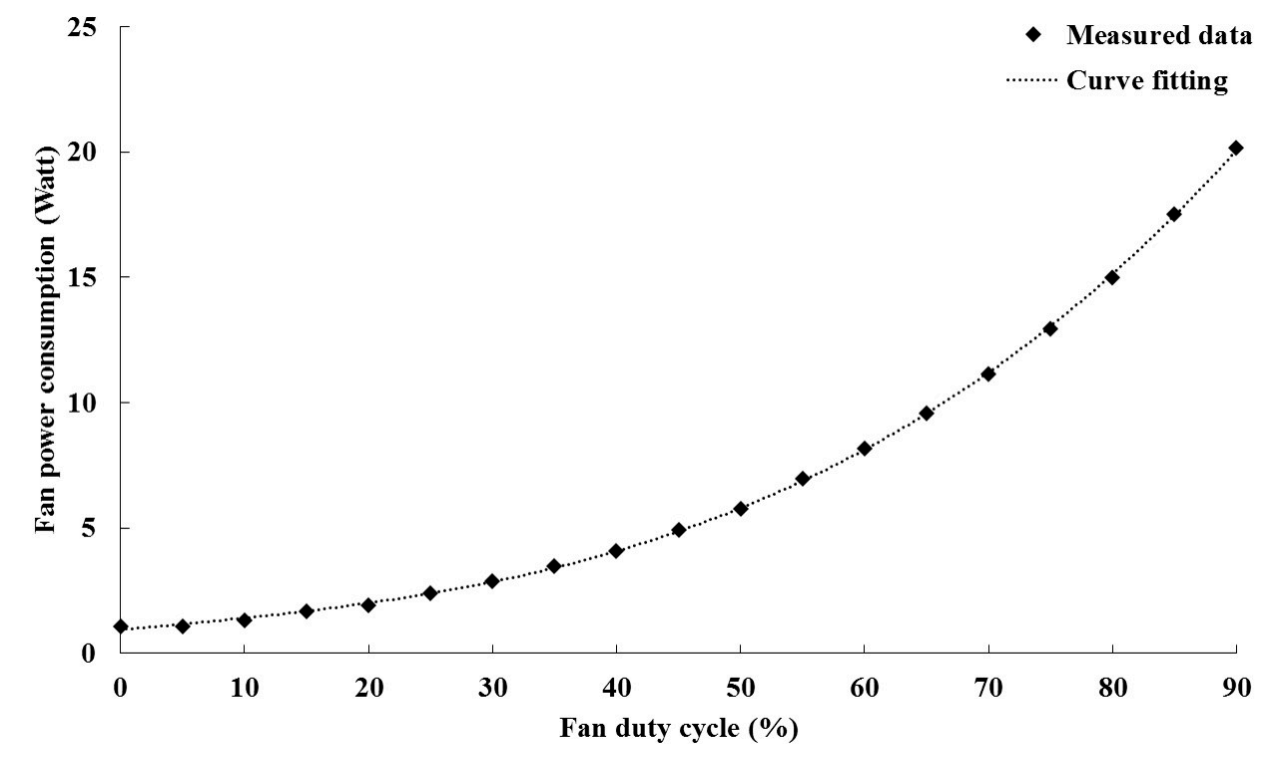
Fan power model.

In this study, we chose the Nidec Ultraflo R40W12BS5AC-65 “Double Wide” 4CM fan as the air mover, and its rating is 0.8 A at 12 V. Two fans are grouped into a one-fan zone. The PWM driving signal between 0% and 100% is injected into the fan with an increment of 5% per sample. Given the fan duty cycle from 0% to 100%, the fan runs from idle to maximum speed and the fan power is correspondingly measured. Figure 2 shows the relationship between the fan power and fan speed of the fan zone. Because of the saturation of the fan speed at 90%, the data at 95% and 100% are ignored for a more accurate fitting curve. Consequently, the constant coefficients are obtained through the third-order nonlinear curve fittings, which are a = 2e−5, b = 2.6e−5, c = 0.045, and d = 0.94.

## 3. Design of the Optimal Self-Tuning PID Controller for the Server Fan Cooling System

**Figure 3:**
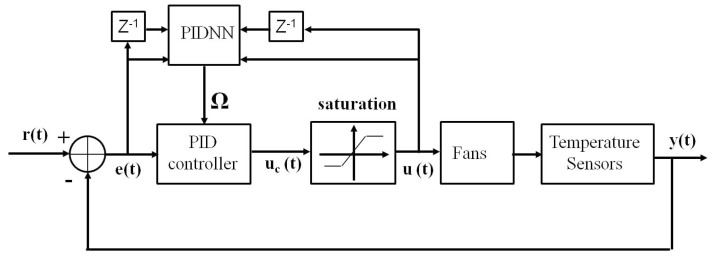
The block diagram of the self-tuning proportional-integral-derivative (PID) control system.

In this section, the PID controller and the online learning algorithm of the neural network are derived. The block diagram of the self-tuning PID control system is shown in Figure 3. The relative symbols are defined as follows:

*r*(t): Temperature set-points
*y*(t): Output of temperature sensors
*e*(t): Errors of the output temperatures and set-points
*u*(t): fan speed
Ω = [PID]: PID control gains

The control performance depends on the combination of PID gains. Although a suitable combination of the three gains can be obtained through trial and error by a skilled engineer to make the system stable, fixed PID coefficients are most suitable for power efficiency. Comparatively, manual trial-and-error PID tuning consumes excessive labor and time. Therefore, in this study, a PIDNN tuning method was proposed, and the optimization was defined to minimize the fan power consumption during transient response time.

(3) $$\int_{t_{1}}^{t_{2}}P(F)\cdot u(t)dt$$

where the interval between *t*1 and *t*2 implies one cycle of the transient response time.

### 3.1. PID Controller

In general, the transfer function of a PID controller is as follows:

(4) $$G_{c}\text{(}s\text{)}=K_{p}+\frac{K_{i}}{s}+K_{d}s$$

Thus, the controller can be described as:

(5) $$u\text{(}t\text{)}=u_{0}+K_{c}\text{[}e\text{(}t\text{)}+\frac{1}{\tau_{i}}\int_{0}^{t}e\text{(}t\text{)}dt+\tau_{d}\frac{de\text{(}t\text{)}}{dt}\text{]}$$

where *u_0_* is the control bias of the PID controller and *e*(*t*) = *r*(*t*) − *y*(*t*) is the error of the measured output temperature *y*(*t*) and the temperature set-point *r*(*t*). The variable *K_c_* is a constant gain, and *τ_i_* and *τ_d_* are integral and derivative time, respectively. The derivative action in Equation (5) will cause the derivative-kick because the sudden step change in error signal results in the derivative of the error to be infinite. We express the derivative term as follows:

(6) $$\frac{de\text{(}t\text{)}}{dt}=\frac{dr\text{(}t\text{)}}{dt}-\frac{dy\text{(}t\text{)}}{dt}$$

Under normal operation, the set-point is assumed to be constant. Hence, Equation (6) can be modified to:

(7) $$\frac{de\text{(}t\text{)}}{dt}=-\frac{dy\text{(}t\text{)}}{dt}$$

Therefore, the derivative action is based on *y*(*t*) rather than *e*(*t*), and the derivative-kick is eliminated. From Equations (5) to (7), the PID controller, at an instant time *t*, in discrete form can be written as follows:

(8) $$u\text{(}t\text{)}=u_{0}+K_{c}\text{[}e\text{(}t\text{)}+\frac{\text{Δ}t}{\tau_{i}}\sum_{i=1}^{n}e\text{(}i\text{Δ}t\text{)}-\tau_{d}\frac{y\text{(}t\text{)}-y\text{(}t-\text{Δ}t\text{)}}{\text{Δ}t}\text{]}$$

where *n* is the number of sampling points, and Δ*t* is the sampling time. At *t* − Δ*t*, Equation (8) can be expressed as follows:

(9) $$u\text{(}t-\text{Δ}t\text{)}=u_{0}+K_{c}\text{[}e\text{(}t-\text{Δ}t\text{)}+\frac{\text{Δ}t}{\tau_{i}}\sum_{i=1}^{n-1}e\text{(}i\text{Δ}t\text{)}-\tau_{d}\frac{y\text{(}t-\text{Δ}t\text{)}-y\text{(}t-2\text{Δ}t\text{)}}{\text{Δ}t}\text{]}$$

By subtracting Equations (9) from (8), the velocity form of the PID controller can be written as follows:

(10) $$\begin{array}{l} \text{Δ}u\text{(}t\text{)}=K_{c}\text{[(y(}t-\text{Δ}t\text{)}-\text{y(t))}+\frac{\text{Δ}t}{\tau_{i}}e\text{(}t\text{)}-\tau_{d}\text{(}\frac{y\text{(}t\text{)}-2y\text{(}t-\text{Δ}t\text{)}+y\text{(}t-2\text{Δ}t\text{)}}{\text{Δ}t}\text{)]}\\ \;=K_{p}\text{(y(}t-\text{Δ}t\text{)}-\text{y(t))}+K_{i}e\text{(}t\text{)}+K_{d}\text{(}-y\text{(}t\text{)}+2y\text{(}t-\text{Δ}t\text{)}-y\text{(}t-2\text{Δ}t\text{))} \end{array}$$

Three separate parameters involved in Equation (10) are the proportional (*K_p_*), integral (*K_i_*), and derivative (*K_d_*) constants. They are the controller gains, *P*, *I*, and *D*, respectively, which will be adjusted by PIDNN. The velocity form of the PID controller has two advantages: (a) The value *u_0_* is eliminated and (b) No overflow error occurs because the summation term is eliminated. Finally, after setting the sampling time, Δ*t* = 1 s, the fan speed can be calculated using the following equation, where *u*(*t* + 1) is the new fan speed and *u*(*t*) is the current fan speed.

(11) $$\begin{array}{ll} u\text{(}t+1\text{)} & =u\text{(}t\text{)}+\text{Δ}u\text{(}t\text{)}\\ \; & =u\text{(}t\text{)}+K_{p}\text{(}y\text{(}t-1\text{)}-y\text{(}t\text{))}+K_{i}\text{(}r\text{(}t\text{)}-y\text{(}t\text{))}+K_{d}\text{(}-y\text{(}t\text{)}+2y\text{(}t-1\text{)}-y\text{(}t-2\text{))} \end{array}$$

### 3.2. PID Self-Tuning

The main concept of PIDNN was proposed by Shu [22]. It is a specific kind of networks, which is strictly designed to be a three-layer structure. Moreover, the activation function defined in its hidden layer neurons is simply work as PID controller. We take the advantage of the PIDNN combining the PID control and explicit neural structure, and then the weights are adjusted using the back-propagation (BP) algorithm. To guarantee the convergence of tracking error, a discrete-type Lyapunov function was analyzed and derived to train the PIDNN effectively [23]. When using BP adaptive law, a sign function is typically used because of the lack of the transfer function in the system. However, this may result in a singular point in time-delayed systems such as the fan-based cooling system. Therefore, a modified sign function is proposed to avoid the possibility of the singular point.

**Figure 4:**
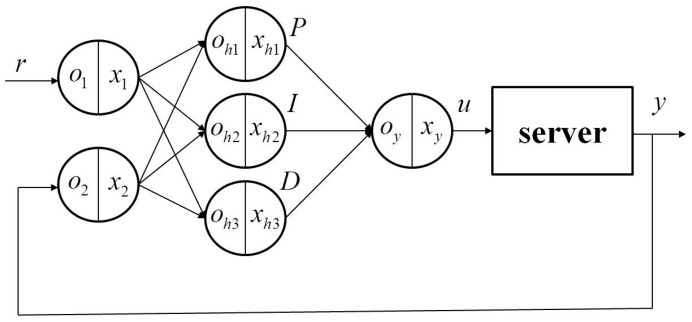
Structure of PIDNN for executing an online self-tuning PID controller for a server.

The PIDNN structure used for executing online PID self-tuning of the server is shown in Figure 4, where *r* is the set-point, *u* is the fan speed, and *y* is the output temperature. The network is a three-layer structure, comprising the input layer, hidden layer, and output layer. The symbols *o* and *x* of each neuron represent the input and output of each neuron respectively, and the suffixes of *o* and *x* correspond to each layer. The input layer has two neurons, which are designed for error measurement, receiving the set-point and feedback temperature, and then generating the error signal to the hidden layer. The weighting from the input layer to the hidden layer has preset values of *w_11_* = *w_12_* = *w_13_* = 1 and *w_21_* = *w_22_* = *w_23_* = −1. The hidden layer consists of *P*, *I*, and *D* neurons, which can execute the PID control algorithms. One neuron in the output layer adds the product of the outputs of the hidden layer and their corresponding weighting values, which are *w_1o_*, *w_2o_*, and *w_3o_*, representing the constants of PID. Therefore, the PID constants are updated online to meet the requirements of the temperature response of the server by using BP algorithm.

The goal of the PID auto-tuning is to select the suitable combination of PID constants by developing an algorithm such that each temperature response of five components has the requirements of minimal overshoot and low-amplitude oscillation. This study used integral-square-error (ISE) criterion for the suitable combination of PID gains, which can be expressed as:

(12) $$ISE=\int_{0}^{\infty }e^{2}\text{(}t\text{)}dt$$

where the error *e* represents the difference between the set-point and output temperatures. To minimize the ISE by adjusting the PID gains online, the cost function is defined as:

(13) $$J\text{(}m\text{)}=\frac{1}{2}\sum_{m=1}^{n}e^{2}\text{(}m\text{)}$$

where *m* is the number of sampling points with a 1 s sampling period and *n* is the total number of sampling points. The following updated BP law was used:

(14) $$w_{jo}\text{(}m\text{)}=w_{jo}\text{(}m-1\text{)}-\eta \frac{\partial J}{\partial w_{jo}\text{(}m-1\text{)}}\;(j=1,2,3)$$

where *η* is the learning coefficient that determines the adjusting resolution of PID gains. According to the chain rule, the partial derivative term of Equation (14) can be denoted as follows:

(15) $$\frac{\partial J}{\partial w_{j0}}=\frac{\partial J}{\partial e}\frac{\partial e}{\partial y}\frac{\partial y}{\partial u}\frac{\partial x_{y}}{\partial o_{y}}\frac{\partial o_{y}}{\partial w_{j0}}$$

where $\partial o_{y}/\partial w_{jo}$ implies the outputs of the hidden layer, which are *x_h1_*, *x_h2_*, and *x_h3_*. The active functions of the three neurons in the hidden layer are proportional, integral, and derivative actions, respectively. Therefore, the relationship between the input and output of the hidden layer can be written as:

(16) $$x_{h1}\text{(}m\text{)}=\left\{ \begin{array}{l} 1,\;o_{h1}\text{(}m\text{)}>1\\ o_{h1}\text{(}m\text{)},\;-1\leq o_{h1}\text{(}m\text{)}\leq 1\\ -1,\;o_{h1}\text{(}m\text{)}<-1 \end{array}\right.\;$$

(17) $$x_{h2}\text{(}m\text{)}=\left\{ \begin{array}{l} 1,\;o_{h2}\text{(}m\text{)}>1\\ o_{h2}\text{(}m\text{)}+o_{h2}\text{(}m-1\text{)},\;-1\leq o_{h2}\text{(}m\text{)}\leq 1\\ -1,\;o_{h2}\text{(}m\text{)}<-1 \end{array}\right.$$

(18) $$x_{h3}\text{(}m\text{)}=\left\{ \begin{array}{l} 1,\;o_{h3}\text{(}m\text{)}>1\\ o_{h3}\text{(}m\text{)}-o_{h3}\text{(}m-1\text{)},\;-1\leq o_{h3}\text{(}m\text{)}\leq 1\\ -1,\;o_{h3}\text{(}m\text{)}<-1 \end{array}\right.$$

All the inputs of the hidden layer are designed as the difference between the set-point and feedback temperatures and are expressed as follows:

(19) $$o_{h1}\text{(}m\text{)}=e\text{(}m\text{)}$$

(20) $$o_{h2}\text{(}m\text{)}=e\text{(}m\text{)}$$

(21) $$o_{h3}\text{(}m\text{)}=e\text{(}m\text{)}$$

For the server fan cooling system, ∂y/∂u in Equation (15) cannot be obtained because the system model of the server is unknown. In general, the sign function replaces the actual value of ∂y/∂u, as follows:

(22) $$\frac{\partial y}{\partial u}=s\;gn\text{(}\frac{y\text{(}m\text{)}-y\text{(}m-1\text{)}}{u\text{(}m\text{)}-u\text{(}m-1\text{)}}\text{)}$$

However, *u* is the fan speed that may not change in the sampling period. Consequently, the denominator of Equation (22) becomes zero, which causes an unexpected singular point. Practically, the sign function of ∂y/∂u implies a change in the direction of the control input and system output. For instance, if the input value increases and the output value decreases, then ∂y/∂u = −1. Conversely, if both the input and output values increase, then ∂y/∂u = 1. Therefore, to avoid generating a singular point, Equation (22) is modified as:

(23) $$\frac{\partial y}{\partial u}=s\;gn\text{[(}y\text{(}m\text{)}-y\text{(}m-1\text{))}\times \text{(}u\text{(}m\text{)}-u\text{(}m-1\text{))]}$$

## 4. Experiments and Results

**Figure 5:**
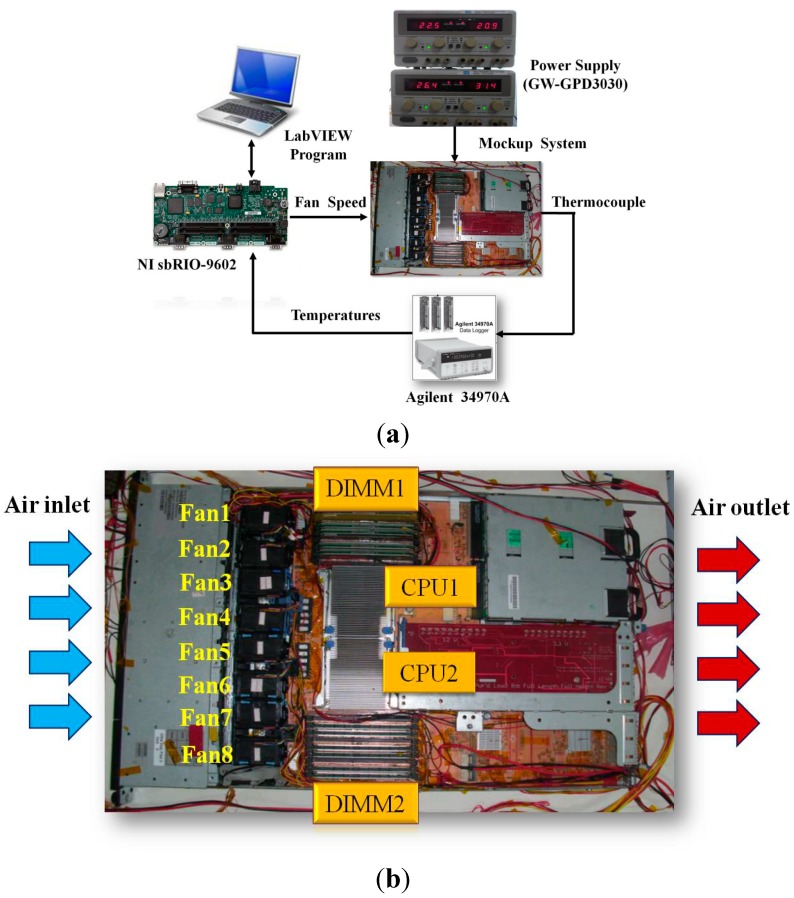
(**a**) Configuration of the server fan control system. Add a descriptive label of the figure here; (**b**) Server mockup system.

The server fan control system, as illustrated in Figure 5a, was established to explore fan power saving by an optimally tuned PID control. Figure 5b shows the image of the server mockup system. The hardware and instrumentation used in this system include the power supply-based heater, NI sbRIO-9602 embedded control and data acquisition PC Board, Agilent 34970A Data Logger, and LabVIEW software installed on a Windows-based PC. Here, the power supply (GW-GPD3030) was used to provide thermal loading to the electronic components that can simulate the heat behavior of server operation. Numerous temperature sensor thermocouples were mounted on the electronic components. When the components were heated, the Data Logger acquired the temperatures through the sensors. Through LabVIEW GUI programming, a control algorithm was implemented and the fan speed output temperatures were acquired. The program was loaded into the NI sbRIO-9602, which integrates a real-time processor, a user-reconfigurable field-programmable gate array (FPGA), and input and output (I/O) ports. The NI sbRIO-9602 then collected temperatures from the Data Logger and generated the PWM signal through FPGA to adjust the fan speed, and the temperature response was monitored by the PC.

This study employed zone-based cooling strategy. Eight parallel mounted fans were grouped into Fans 1 and 2 for cooling DIMM1, Fans 3 and 4 for cooling CPU1, Fans 5 and 6 for cooling CPU2, and Fans 7 and 8 for cooling DIMM2. To control the fan speed, PID controllers were used, and the temperature sensors provided the feedback signal to the controllers. The fans adjusted the airflow rate to correspond to the rise or fall in temperature that was maintained by the components at their own temperature set-point. Each component should be given three PID gains and a temperature set-point. Thus, this server fan cooling system required four PID gain combinations.

**Figure 6:**
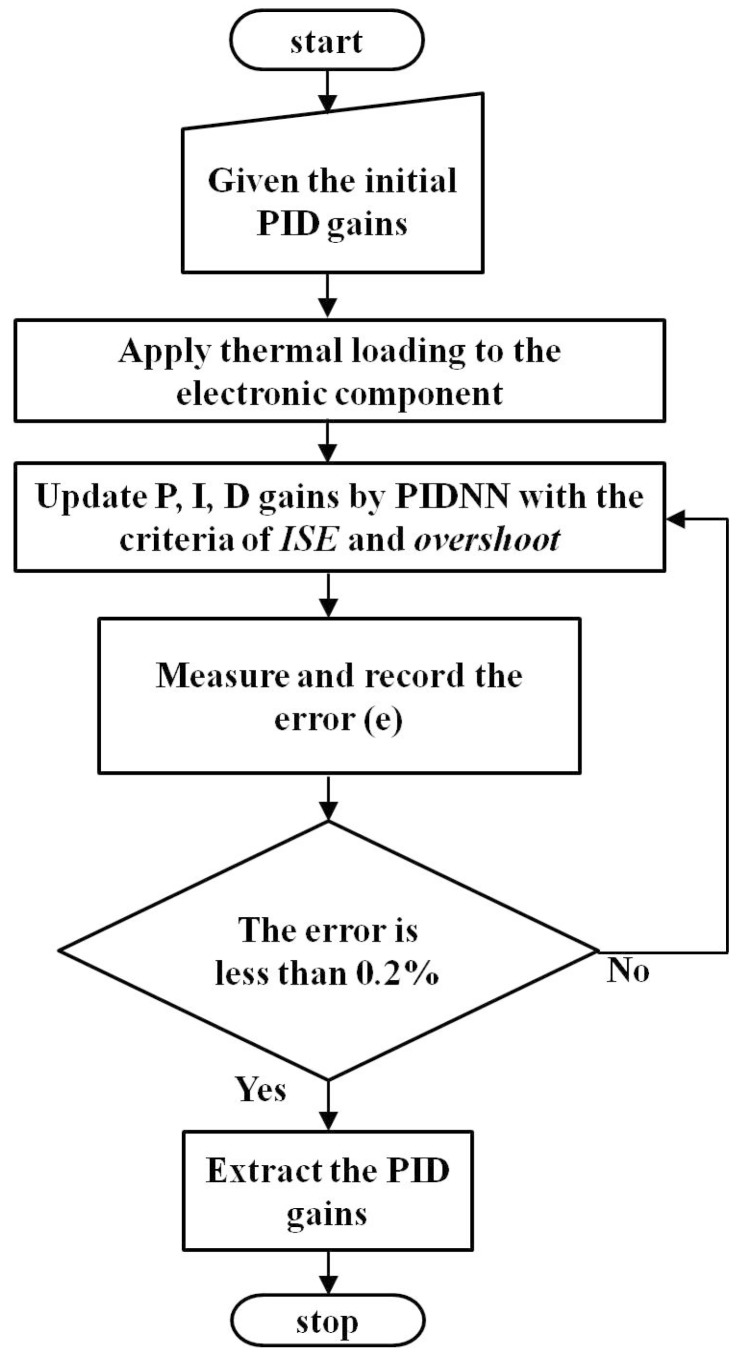
The flow chart of PID self-tuning.

**Figure 7:**
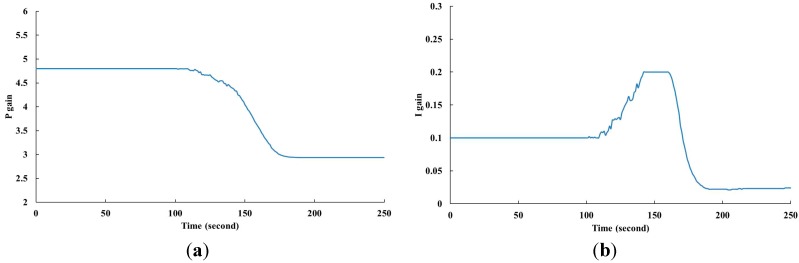

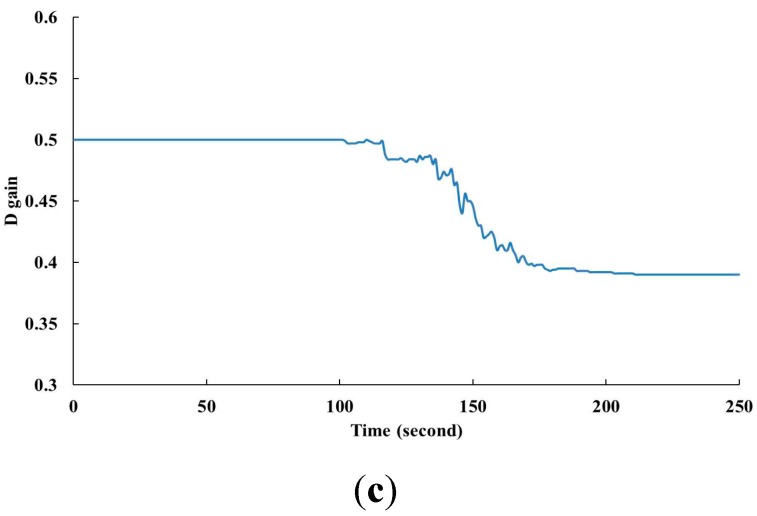
The result of PID self-tuning. (**a**) The process of P gain; (**b**) The process of I gain; (**c**) The process of D gain.

To validate the proposed method, we chose CPU1 as the representative component to discuss the effect of various time domain specifications on fan power consumption. Thermal loading of 80 W was provided to CPU1 and the temperature set-point was 55 °C. The flow chart of the PID self-tuning process is shown in Figure 6. First, the initial values of PID gains should be obtained, and it was easily to estimate them through the input-output relationship. A step change of fan speed was applied, and then the initial values of PID gains could be obtained from the temperature response. The initial PID gains were updated by PIDNN with the criteria of ISE and overshoot. The updating law used in this work is to minimize the ISE by adjusting the PID gains on-line. Besides, the criterion of overshoot was included to explore the fan power consumption. When the measured error was less than 0.2%, stop the tuning process and the combination of PID gains was obtained. The result of PID self-tuning is shown in Figure 7. It consumed about 200 s to tune the PID gains.

**Figure 8:**
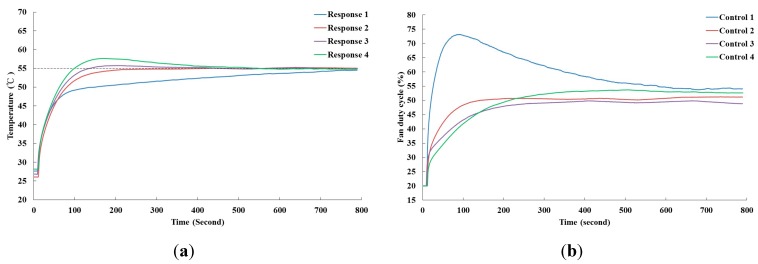
Control results of various PID controllers for CPU1. (**a**) The temperature responses of CPU1 by the PID controller; (**b**) The control efforts of Fans 3 and 4 by the PID controller.

Figure 8 shows the control results of four PID controllers tuned by PIDNN with different time domain specifications for CPU1. From Figure 8a, it is observed that the temperatures of the four responses eventually converge to the set-point. Practically, these controllers were suitable for the server fan cooling system because the temperature can be maintained at the set-point. However, the criterion of the transient-state temperature response in the time domain considerably affects the control effort that superior fan power efficiency can be obtained by using the appropriate criterion. Obviously, the output of Controller 1 consumes more power than that of the other controllers during the time interval of 800 s because the design of Controller 1 initially generates high control force resulting in a slow convergence of Response 1 compared with the other responses. Table 1 lists the combinations of the four PID coefficients and their corresponding fan power consumption, which are calculated using Equation (3). In this work, we focused on the effect of temperature response on fan power consumption, and found the major cause is the criterion overshoot. P gain and I gain are the components to decide the value of the overshoot. If we only change I and fix the other two parameters, an approximately response can be obtained as the results with P gain changed and I and D are fixed in this work. The responses, obtained from either changing P or I gain, produce the approximate fan outputs and the power consumption. Therefore, the I and D gains are fixed on purpose such that it is easily implemented in practical for only adjusting P gain. A minor overshoot can be observed as Response 3 slowly increases the control force resulting in lower fan power consumption. However, an excessive overshoot such as the result of Response 4, which consumes more power than Response 3 because the effort of Control 4 exceeds that of Control 3 after 200 s to suppress the temperature of Response 4. Therefore, the results suggest that it is more advantageous to permit a slight overshoot of the temperature response when designing the fan speed controller.

**Table 1:** Four PID controllers in various time domain specifications and the power consumption of Fans 3 and 4.

	P	I	D	Overshoot	Fan Power (W)
Controller 1	2.854	0.021	0.389	non	6422.78
Controller 2	1.854	0.021	0.389	non	4463.02
Controller 3	1.354	0.021	0.389	1.38%	4147.13
Controller 4	0.854	0.021	0.389	4.83%	4566.20

As mentioned, the PID gains of the four electronic components are obtained through PIDNN self-tuning with the criterion of overshoot of 1%–3.5%. In fact, different controller gains may result in different transient responses including overshoot, and rise and settling time. In the case of fan cooling system, it was found that the main impact on fan power is the criterion of temperature overshoot, and the other two criteria are proportional to the overshoot. For example, the less the rise time brings to the more the overshoot. Therefore, we focused on the temperature overshoot to discuss the fan power consumption. The power load conditions of the components and their corresponding temperature set-points are given as follows:

CPU1: The power load is 80 W and the set-point is 55 °C.
CPU2: The power load is 80 W and the set-point is 57.5 °C.
DIMM1: The power load is 60 W and the set-point is 62.5 °C.
DIMM2: The power load is 60 W and the set-point is 60 °C.

**Figure 9:**
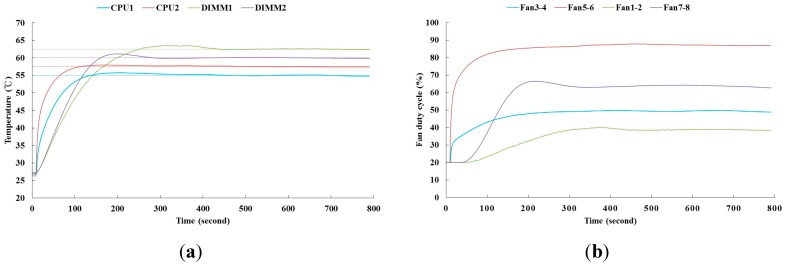
Control results of PID controllers with an overshoot. (**a**) The temperature responses of the electronic components by the PID controllers; (**b**) The control efforts of the fans by the PID controllers.

**Figure 10:**
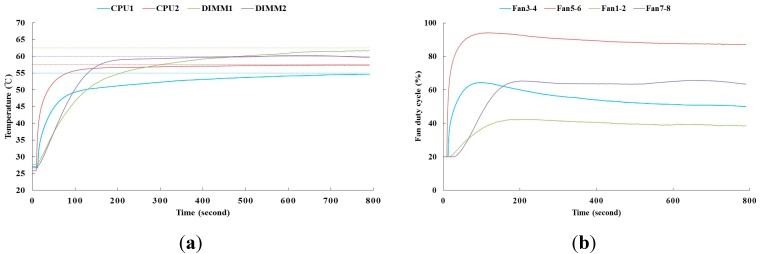
Control results of PID controllers without an overshoot. (**a**) The temperature responses of the electronic components by the PID controllers; (**b**) The control efforts of the fans by the PID controllers.

Figure 9 shows the result of the fan speed control by the proposed PID controller for the study’s constructed server. To compare the fan power efficiency, the PID controller results without the temperature response overshoot is shown in Figure 10. In our system, it needs time to response the rising temperature, read by the sensors, while the thermal loading is applied to electronic components. Therefore, the time delayed is seen as shown in Figure 9 and Figure 10. The results of the proposed method indicate that each control effort of the fan consumes less power during the transient time of 800 s. Table 2 and Table 3 show the corresponding PID gains and fan power consumption. It appears that fan power efficiency is more dependent on the transient-state temperature response of the electronic components. In this study, up to 14% of fan power was saved at the time interval of 800 s by the proposed power-based optimization self-tuning PID controller while the 1U rack server operates from the idle to peak power state.

**Table 2:** PID gains with overshoot and fan power consumption.

	P	I	D	Overshoot	Fan Power (W)
CPU1	1.354	0.021	0.389	1.38%	4174.13
CPU2	2.854	0.048	0.611	1.1%	13,589.56
DIMM1	2.064	0.021	0.507	3.3%	6353.39
DIMM2	3.747	0.045	0.499	1.76%	2736.95
Total Fan Power					26,854.03

**Table 3:** PID gains without overshoot and fan power consumption.

	P	I	D	Fan Power (W)
CPU1	2.854	0.021	0.389	6422.78
CPU2	3.254	0.038	0.611	15,148.52
DIMM1	2.164	0.011	0.507	6550.55
DIMM2	4.747	0.035	0.499	3087.87
Total Fan Power				31,209.72

## 5. Conclusions

This study applied a PIDNN with the time domain criterion, based on low fan power consumption, to develop an optimal self-tuning PID controller for a server cooling system. A server mockup system was constructed to simulate a 1U rack server, and a fan power model was created to explore the fan power efficiency by fan speed control. On the basis of the nonlinear learning ability characteristic of the neural network, PIDNN was used to tune all online and optimized PID gains. The optimal tuning is based on low fan power consumption during the temperature transient response of the server’s electronic components. In order to mimic the thermal loading of an operating server, power loads and temperature set-points were provided to the electronic components, and then the step response was tested using the proposed controllers. Firstly, CPU1 was used as the representative component to realize how the controller design affects on the fan power consumption. Then we compared the fan power consumption of various PID controllers on the basis of overshoot criteria in the time domain. Fan power usage can be more efficiency by well controlled the transient-response of the temperature to allow a little overshoot. Finally, using the same way as PID tuning for CPU1, the PID controllers of the four components were obtained. The results show that a slight temperature response overshoot saves more fan power than no overshoot. The contributions of this work can be concluded as follows:

(1) This is the first study that discusses how the controller affects fan power consumption in the transient time when the server operates from low to high power state. This method may result in more fan power saving, comparing to the previous researches that focused on the steady-state solution.
(2) Without complicated modeling of the server cooling system, fan power efficiency can be improved by the proposed method.
(3) In general, a skilled engineer may cost a number of hours to tune a PID controller. The results of this work suggest that the proposed controller is not only more suitable for fan power efficiency but also saves considerable labor and time when tuning the PID gains.
(4) During the transient time of 800 s, it appears that up to 14% of fan power can be saved for the 1U rack server when the overshoot criteria falls between 1% and 3.5% in the design of a self-tuning PID controller.

## References

[1] Lefurgy C., Rajamani K., Rawson F., Felter W., Kistler M., Keller T. (2003). Energy Management for Commercial Servers. IEEE Comput..

[2] Patel C.D., Bash C.E., Sharma R., Beitelmam M., Friedrich R.J. Smart Cooling of Data Centers. Proceedings of the Pacific Rim/ASME International Electronic Packaging Technical Conference and Exhibition.

[3] Greenberg S., Mills E., Tschudi B., Rumsey P., Myatt B. Best Practices for Data Centers: Lessons Learned from Benchmarking 22 Data Centers. Proceedings of the ACEEE Summer Study on Energy Efficiency in Buildings.

[4] Chiueh H., Luh L., Draper J., Choma J. A Novel Fully Integrated Fan Controller for Advanced Computer Systems. Proceedings of the Southwest Symposium on Mixed-Signal Design.

[5] Wang Z., Bash C., Tolia N., Marwah M., Zhu X., Ranganathan P. Optimal Fan Speed Control for Thermal Management of Servers. Proceedings of the ASME/Pacific Rim Technical Conference and Exhibition on Packaging and Integration of Electronic and Photonic Systems, MEMS, and NEMS.

[6] Rajamani K., Rawson F., Ware M., Hanson H., Carter J., Rosedahl T., Geissler A., Silva G., Hua H. Power-Performance Management on an IBM POWER7 Server. Proceedings of the 2010 ACM/IEEE International Symposium on Low-Power Electronics and Design (ISLPED).

[7] Han X., Joshi Y. Energy Reduction in Server Cooling via Real Time Thermal Control. Proceedings of the 28th IEEE Semiconductor Thermal Measurement and Management Symposium.

[8] Astrom K.J., Hagglund T. (2001). The Future of PID Control. Control. Eng. Pract..

[9] Ntogramatzidis L., Ferrante A. (2011). Exact Tuning of PID Controllers in Control Feedback Design. IET Control Theory Appl..

[10] Yamamoto T., Takao K., Yamada T. (2009). Design of a Data-Driven PID Controller. IEEE Trans. Control Syst. Technol..

[11] Wang L., Du W., Wang H., Wu H. (2008). Fuzzy Self-Tuning PID Control of the Operation Temperatures in a Two-Staged Membrane Separation Process. J. Nat. Gas. Chem..

[12] Zeng S., Hu H., Xu L., Li G. (2012). Nonlinear Adaptive PID Controller for Greenhouse Environment Based on RBF Network. Sensors.

[13] Shu H.L., Pi Y. (2000). PID Neural Networks for Time-Delay Systems. Comput. Chem. Eng..

[14] Chen J.H., Huang T.C. (2004). Applying Neural Networks to on-line Updated PID Controllers for Nonlinear Process Control. J. Process. Control..

[15] Ren T.J., Chen T.C., Chen C.J. (2008). Motion Control for a Two-Wheeled Vehicle Using a Self-Tuning PID Controller. Control. Eng. Pract..

[16] Cong S., Liang Y. (2009). PID-Like Neural Network Nonlinear Adaptive Control for Uncertain Multivariable Motion Control Systems. IEEE Trans. Ind. Electron..

[17] Chen S.Y., Lin F.J. (2013). Decentralized PID Neural Network Control for Five Degree-of-Freedom Active Magnetic Bearing. Eng. Appl. Artif. Intell..

[18] Liu G.P., Daley S. (2001). Optimal-Tuning PID Control for Industrial Systems. Control. Eng. Pract..

[19] Skadron K., Stan M., Huang W., Velusamy S., Sankaranarayanan K., Tarjan D. Temperature-Aware Microarchitecture: Modeling and Implementation. Proceedings of the 30th Annual International Symposium on Computer Architecture.

[20] Ayoub R., Indukuri K., Rosing T.S. (2011). Temperature Aware Dynamic Workload Scheduling in Multisocket CPU Servers. IEEE Trans. Comput. Aided Design Integr. Circuits Syst..

[21] Patterson M. The Effect of Data Center Temperature on Energy Efficiency. Proceedings of the 11th Intersociety Conference on Thermal and Thermomechanical Phenomena in Electronic Systems.

[22] Shu H.L. (1997). Study on the Neural PID Network Based Cascade Control System. Autom. Instrum..

[23] Lin F.J., Hwang J.C., Tan K.H., Lu Z.H., Chang Y.R. (2012). Intelligent Control of Doubly-Fed Induction Generator Systems Using PIDNNs. Asian J. Control.

